# Intermittent Fever and Atmospheric Temperature

**Published:** 1889-11

**Authors:** 


BUFFALO MEDICAL AND SURGICAL JOURNAL
A MONTHLY REVIEW OF MEDICINE AND SURGERY.

*EDITORS:*
THOS. LOTHROP, M. D. - - - W. W. POTTER, M. D.
All communications, whether of a literary or business character, should be addressed to the editors: 284 FRANKLIN STREET, BUFFALO, N. Y.

### Editorial.

### INTERMITTENT FEVER AND ATMOSPHERIC TEMPERATURE.

IN SOME interesting studies of intermittent fever and its relations to atmospheric temperature recently made by Dr. Henry B. Baker, Secretary of the Michigan State Board of Health, he arrives at the following conclusions :

1. Intermittent fever is proportional, directly or inversely, to the average daily range of atmospheric temperature.

2. The controlling cause of intermittent fever is exposure to insidious changes, or changes to which one is unaccustomed, in the atmospheric temperature.

3. In the mechanism of the causation of intermittent fever, the chief factor is the delay in the reaction from exposure to cool air ; this delay, extending to a time when greater heat loss should occur, results in the abnormal accumulation of heat in the interior of the body, and in disturbing nervous action—the chill ; and the final reaction is excessive because of the accumulation of heat, and sometimes because it occurs at the warmest part of the day.

4. The fever is the excessive reaction from the insidious influence of exposure to cool air ; and it is periodical because of the periodicity of nervous action, and because the exposure and consequent chill are periodical, owing to the nightly absence of the warmth of the sun.

5. Residence in valleys or on low lands through which, or upon which, cold air floats at night, and thus causes insidious changes in the atmospheric temperature, favor intermittent fever.

6. In our climate, those measures (such as drainage) which enable the soil to retain the warmth during the night, and thus reduce the daily range of temperature immediately over such soil, tend to reduce intermittent fever among the residents thereon.

7. In the cure and prophylaxis of intermittent fever, those remedies are useful which lessen torpidity and tend to increase the power of the body to react promptly to insidious changes in the atmospheric temperature.

8. The slowness of the pulse, and other indications of torpidity associated with retention of bile, or with certain disturbances of the function of the liver, are well known; but, so far as known to the writer, these conditions have not hitherto been considered as causative of the fever in the manner herein suggested.

These conclusions may be in perfect keeping with meteorological observations, but when considered from a pathological standpoint certain discrepancies are encountered. Certain atmospheric changes, under certain conditions, *do* effect the onset of an attack of intermittent fever; but, in such cases, the causative agent may be sought for in the blood of the individual rather than in the surrounding media. The action of excessive changes of temperature is instrumental in the production of miasmatic poisons which enter the system, and, in turn, as the system yields or not, engender an attack of intermittent fever.

The researches of Laverain in Algiers, communicated to the French Academy of Medicine, in 1881 and 1882, go to show that in the blood of malarial patients certain characteristic elements are present. He found (1) crescentic pigmented bodies; (2) pigmented bodies in the interior of red blood corpuscles; and (3) pigmented flagellate organisms. These observations have been many times confirmed, especially by Richard, Marchiafava, Celli, Councilman, and others, and the organism designated as "plasmodium malariæ." Osler has recently made very extensive enquiries into the character of this organism, and found them (being polymorphic) in sixty-two out of seventy cases examined. Failure to find them in the other eight cases he believes to be due to some source of error.

It is more plausible to infer that these organisms, or their ptomaines, by entering the general circulation, produce their characteristic poisoning effects on the brain centers, and especially on the thermal centers, than to consider the fever as "the excessive reaction from the insidious influence of exposure to cool air."

The explanation offered for the periodicity of the attacks of intermittent fever as "owing to the nightly absence of the warmth of the sun," could explain the quotidian type, but could hardly be accepted in those cases where the double quotidian, tertian, or quartan types exist

The writer further says that "in the mechanism of the causation of intermittent fever, the chief factor is the delay in the reaction from exposure to cool air." In exposure to cool air, the thermotaxic mechanism of the body is brought into play, and by increasing thermogenesis on the one hand, and diminishing thermolysis on the other, the body is kept at a nearly constant fixed temperature. Any disturbance in either of the thermogenetic or thermolytic functions,—and these functions have their centers in the brain,—will produce a disturbance in the thermotaxic mechanism, and it fails to maintain the balance between heat production and heat loss. As a consequence, fever follows—be it intermittent or otherwise designated.

In the cure of intermittent fever, therapeutists have found that those remedies which act on the organisms, act most readily on the fever. The experiments by Osler, on this point, are very conclusive.

